# COVID-19-associated neuroinflammation and astrocyte death in the brain linked to ORF3a-induced activation of Sur1-mediated ion channels

**DOI:** 10.1128/mbio.02012-25

**Published:** 2025-08-13

**Authors:** Volodymyr Gerzanich, Chenyu Zhang, Jiantao Zhang, Bhargava Teja Sallapalli, Shaokai Pei, Mohamed Nasr, Cigdem Tosun, Yanjin Zhang, Qiyi Tang, J. Marc Simard, Richard Y. Zhao

**Affiliations:** 1 Department of Neurosurgery, University of Maryland School of Medicine 12264, Baltimore, Maryland, USA; 2 Department of Pathology, University of Maryland School of Medicine 12264, Baltimore, Maryland, USA; 3 Department of Veterinary Medicine, University of Maryland 1068 , College Park, Maryland, USA; 4 Department of Microbiology, Howard University College of Medicine 12232, Washington, DC, USA; 5 Drug Development and Preclinical Research Branch, Division of AIDS, NIAID, NIH, Bethesda, Maryland, USA; 6 Research & Development Service, VA Maryland Health Care System, Baltimore, Maryland, USA; 7 Department of Microbiology and Immunology, University of Maryland School of Medicine 12264, Baltimore, Maryland, USA; 8 Institute of Human Virology, University of Maryland School of Medicine, Baltimore, Maryland, USA; 9 Institute of Global Health, University of Maryland School of Medicine 12264, Baltimore, Maryland, USA; Washington University in St. Louis School of Medicine, St. Louis, Missouri, USA

**Keywords:** COVID-19, SARS-CoV-2, ORF3a, reactive astrocytes, neuroinflammation and neurotoxicity, Sur1-regulated ion channels, glibenclamide, glycyrrhizin

## Abstract

The coronavirus disease 2019 (COVID-19) pandemic has disproportionately affected individuals with pre-existing medical conditions, such as neurocognitive disorders. Premorbid neurocognitive conditions compounded by COVID-19 can escalate into COVID-associated neurological complications, leading to severe illness or even death. As COVID-19 continues to persist and vaccines lose efficacy against emerging variants, individuals with neurocognitive disorders often experience prolonged symptoms that are further exacerbated by repeated breakthrough infections of highly diversified viral variants due to emergence of new viral mutations. Despite the significance of neurocognitive disorders as risk factors for COVID-19-related mortality and long COVID, the underlying causes remain largely unknown. In this study, we report a link between ORF3a expression and COVID-associated neuroinflammation and neurocytotoxicity in postmortem brain tissues from COVID-19 patients. These findings were further verified through neural cell-based *in vitro* and *in vivo* animal studies introducing ORF3a either alone or in the context of viral infection. As a membrane-associated protein, ORF3a induces upregulation of Sur1-regulated ion channels, resulting in intracellular Ca^2+^ influx, apoptosis, and necrosis through both NF-kB-dependent and independent proinflammatory responses in astrocytes. These findings reveal a novel clinical and mechanistic link between ORF3a and Sur1-regulated ion channels, which are highly responsive to neuroinflammatory conditions causing neurodegeneration. Additionally, we have identified a Food and Drug Administration-approved drug, glibenclamide, and a natural antiviral compound glycyrrhizin that effectively mitigates the neuropathological effects of ORF3a, underscoring the therapeutic potential and clinical significance of these findings.

**IMPORTANCE:**

Coronavirus disease 2019 (COVID-19) disproportionately affects individuals with pre-existing neurocognitive conditions primarily due to COVID-19-associated neuroinflammation and neurotoxicity (CNN), which can progress to COVID-associated neurological disorders (CANDs), leading to severe illness and mortality. Despite CNN’s significant contribution to CANDs and related morbidity and mortality, its underlying causes remain poorly understood. Our study identifies ORF3a as a key driver of CNN, establishing a direct clinical and functional link between ORF3a and CNN linking to CANDs. Mechanistically, ORF3a disrupts ion homeostasis in astrocytes by promoting Ca²^+^ influx through Sur1-regulated ion channels, contributing to CNN. Notably, the Food and Drug Administration-approved drug glibenclamide, a Sur1-specific inhibitor, and the natural compound glycyrrhizin effectively mitigate ORF3a-induced neuropathology, highlighting ORF3a as a promising therapeutic target. These findings present a potential strategy to eliminate CNN and prevent CANDs.

## INTRODUCTION

Coronavirus disease 2019 (COVID-19) caused by SARS-CoV-2 infection has had profound health effects, particularly in individuals with pre-existing medical conditions, including neurocognitive disorders such as dementia, cerebrovascular disease, and post-traumatic stress disorder (1–4). Premorbid neurocognitive conditions compounded by COVID-19 driven by COVID-19-associated neuroinflammation and neurotoxicity (CNN) (5–7) can escalate into COVID-associated neurological disorders (CANDs). CANDs may manifest symptoms ranging from the loss of taste and smell to more severe outcomes, such as stroke and delirium (4, 8). Long-term effects include brain fog, cognitive impairment, and mental health challenges (5, 9). Nationwide cohort studies demonstrated a strong association between CANDs and increased risk of morbidity and mortality in COVID-19 patients (9, 10). A significant percentage of critically ill COVID-19 patients experience CANDs, with up to one-third of cases associated with the severity of the infection or mortality (9–11). Despite the significant impact of CNN on CANDs on COVID-19-related morbidity and long-term outcomes (9, 10), the underlying mechanisms of these complications remain poorly understood.

While SARS-CoV-2 primarily targets the lungs, it also infects other organs, including the brain (12–14), specifically targeting glial cells, including astrocytes (6). COVID-19 can trigger neuroinflammation and neurotoxicity, leading to brain damage, including reduced gray matter (15). Mouse model studies have shown that SARS-CoV-2 infection induces brain damage comparable to that observed in COVID-19 patients (16–18). SARS-CoV-2 activates astrocytes in the infected brain, promoting proinflammatory cytokine production and leading to glial cell death (7). Clinical and animal studies suggest that SARS-CoV-2 infection in the brain leads to CNN, contributing to the development of CANDs. However, the specific viral factors responsible for CNN remain undefined.

Early studies indicate that the viral protein ORF3a exacerbates inflammation and cytotoxicity, contributing to cell and tissue damage (17–22). ORF3a has been implicated in kidney injury in transgenic zebrafish, mice, and patients with acute kidney injury (18, 21) and is associated with neuropathogenesis (17). In animal models, the deletion of *ORF3a* from the viral genome results in reduced lung tissue damage (23, 24), consistent with ORF3a driving these injuries. ORF3a is a presumptive viroporin that exists on the cell membrane as a homodimer or tetramer, with each monomer consisting of 275 amino acids (aa) and a molecular weight (MW) of 31 kDa (25). It features three transmembrane domains with diverse ion channel-related functionalities, including altering cell permeability and ion channels (25–29). ORF3a plays a crucial role in activating the NLRP3 inflammasome (30–32), a major driver of the cytokine storm that is a leading cause of COVID-19-related mortality (9, 31–33).

The sulfonylurea receptor 1 (Sur1)-regulated cation channel plays a critical role in the pathophysiology of acute brain injuries (34, 35). Sur1 encoded by the *Abcc8* gene is a member of the ATP-binding cassette protein superfamily primarily found in the brain, kidney, and pancreatic cells (36). In the brain, the Sur1-Trpm4 channel is minimally expressed under normal conditions but becomes transcriptionally upregulated during neuroinflammation, such as traumatic brain injury (TBI) (37), subarachnoid hemorrhage (SAH) (38), multiple sclerosis/experimental autoimmune encephalomyelitis (MS/EAE) (39, 40), and HIV-1 infection (41). Activation of Sur1-regulated ion channels leads to cell depolarization, Ca²^+^ influx, intracellular edema, and cell death (34, 35).

Glibenclamide (GBC, also known as glyburide), a Food and Drug Administration (FDA)-approved antidiabetic drug, specifically inhibits Sur1 (42). GBC has shown promising results in preclinical and clinical studies for treating various neuroinflammatory conditions associated with Sur1-regulated channels and brain injuries (40, 42–45). In our search for ORF3a inhibitors, we identified glycyrrhizin (GL), a natural compound known for its anti-inflammatory and antiviral properties, including efficacy against SARS-CoV-2 (46–48). We and others have shown that GL not only blocks SARS-CoV-2 replication but also inhibits ORF3a in lung and kidney cells (21, 49).

In this study, we demonstrate a clinical and functional link between ORF3a expression, CNN, and inflammation-induced apoptosis and necrosis of astrocytes in patients with COVID-19. We validated this relationship by examining the neuroinflammatory and neurocytopathic effects of ORF3a expression in glial cells *in vitro*. Additionally, we explored whether a similar association exists in SARS-CoV-2-infected mice by analyzing ORF3a expression and corresponding neuroinflammatory damage in brain tissues of K18-hACE transgenic mice. Finally, we evaluated the therapeutic potential of GBC and GL in mitigating CNN, aiming to counteract the effects of CANDs in affected individuals.

## RESULTS

### Correlation of ORF3a with Sur1 elevation, reactive astrocytes, and neuroinflammation in COVID-19 brain tissue

**Fig 1:**
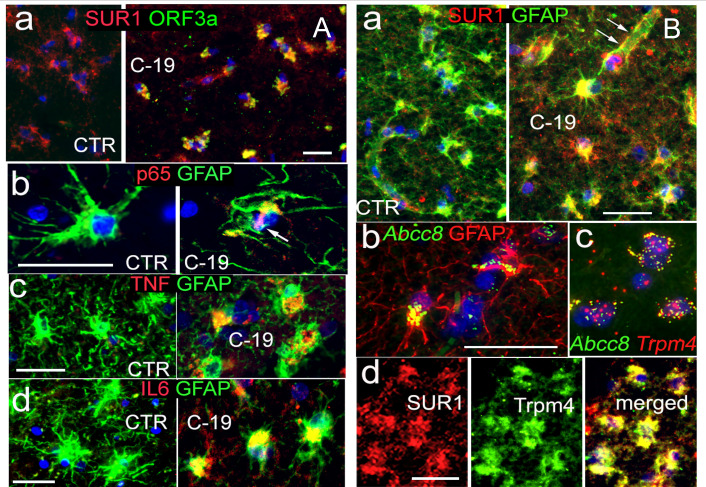
Correlation of ORF3a and Sur1 expression in reactive astrocytes and neuroinflammation in brain tissues of COVID-19 patients. (**A**) Co-immunolabeling of ORF3a and Sur1 in postmortem brain tissue from a COVID-19-positive (C-19) individual (a), along with comparative co-immunostaining of p65 (b), TNF (c), and IL-6 (d) with reactive astrocytes marked by GFAP in COVID-negative control (CTR) and COVID-19-positive (C-19) subjects. (**B**) Co-immunolabeling of Sur1 and GFAP in astrocytes from COVID-19 and CTR subjects (a). Arrows indicate strong expression in astrocytes, including perivascular endfeet. Co-localization of elevated mRNA levels of Abcc8/Sur1 with GFAP (b) and Trpm4 (c) detected using RNAscope, a highly sensitive *in situ* hybridization assay for RNA detection in cells and tissues (56), is further supported by co-immunolabeling of Sur1 and Trmpr4 protein expressions in astrocytes (d). Scale bars are 50 µm. Images are representative of three COVID-19-positive and three CTR cases. Low-resolution images are presented in Fig. S1.

Previous histopathologic examinations of brain tissues from SARS-CoV-2-infected patients with COVID-19 and human brain organoids demonstrated neurodegeneration characterized by neuroinflammation and damage to neurons, astrocytes, and other cells (15, 50–52). However, the virologic causes underlying these neuroinflammatory and neurodegenerative effects remain largely unknown. Given the neuroinflammatory role of Sur1-regulated ion channels in various neuropathologies, we investigated whether a link exists between SARS-CoV-2 ORF3a protein expression, Sur1 expression, and neuroinflammation in COVID-19-affected brain tissues. To explore this potential connection, immunohistochemistry (IHC) was performed on postmortem brain tissues of patients with COVID-19 (Fig. 1). In COVID-negative control (CTR) brain tissues, no immunolabeling was detected with the anti-ORF3a antibody (Fig. 1A–a, left). However, strong immunoreactivity to the anti-ORF3a antibody was observed in COVID-positive (C-19) brain tissues, suggesting specific detection of the ORF3a protein (Fig. 1A–a, right). To validate the viral infection status of ORF3a-positive and control brain tissues, we examined the presence of the viral nucleocapsid (NCp) protein by co-immunostaining with S100B, an astrocytic marker of gray matter (53). NCp protein was clearly detected in C-19 brain tissues but was absent in CTR tissues (Fig. S1). As a structural component of SARS-CoV-2 and a widely used marker of active viral infection (54), the detection of NCp confirms the presence of viral infection in C-19 brain tissues. To provide a broader visualization of ORF3a and NCp in astrocytes of the cortical gray matter from postmortem brain tissues compared to controls, low-resolution images are presented in Fig. S1. Notably, ORF3a expression was closely associated with Sur1 (Fig. 1A–a). Widespread reactive astrocytes indicated by glial fibrillary acidic protein (GFAP) immunopositivity were observed in COVID-positive postmortem brain tissues. Consistent with neuroinflammation, NF-kB (p65) and proinflammatory cytokines, such as TNF and IL-6, were co-localized with reactive astrocytes (Fig. 1A–b–d). Sur1 was elevated in reactive astrocytes in COVID-positive brain tissues but not in COVID-negative controls (Fig. 1B–a). A detailed examination showed strong Sur1 expression in astrocytes, particularly in perivascular endfeet, a specialized subcellular compartment that ensheathes cerebral vasculature and plays crucial roles in maintaining the blood-brain barrier (BBB) (55). Since Sur1-regulated ion channels are activated in response to brain injury through the transcriptional activation of *Abcc8*/Sur1, we used RNAscope, a highly sensitive *in situ* hybridization method (56), to detect *Abcc8* RNA. *Abcc8* RNA was clearly detected in astrocytes and co-localized with its pore-forming partner, Trpm4 (Fig. 1B–b–c). This observation was corroborated by co-immunolabeling of Sur1 and Trpm4 protein expression in astrocytes (Fig. 1B–d). These findings suggest a correlation between ORF3a expression, Sur1-Trpm4 channel expression, and neuroinflammation induced in COVID-19 brain tissues.

### ORF3a induces Sur1 and GFAP upregulation leading to astrocyte death

**Fig 2:**
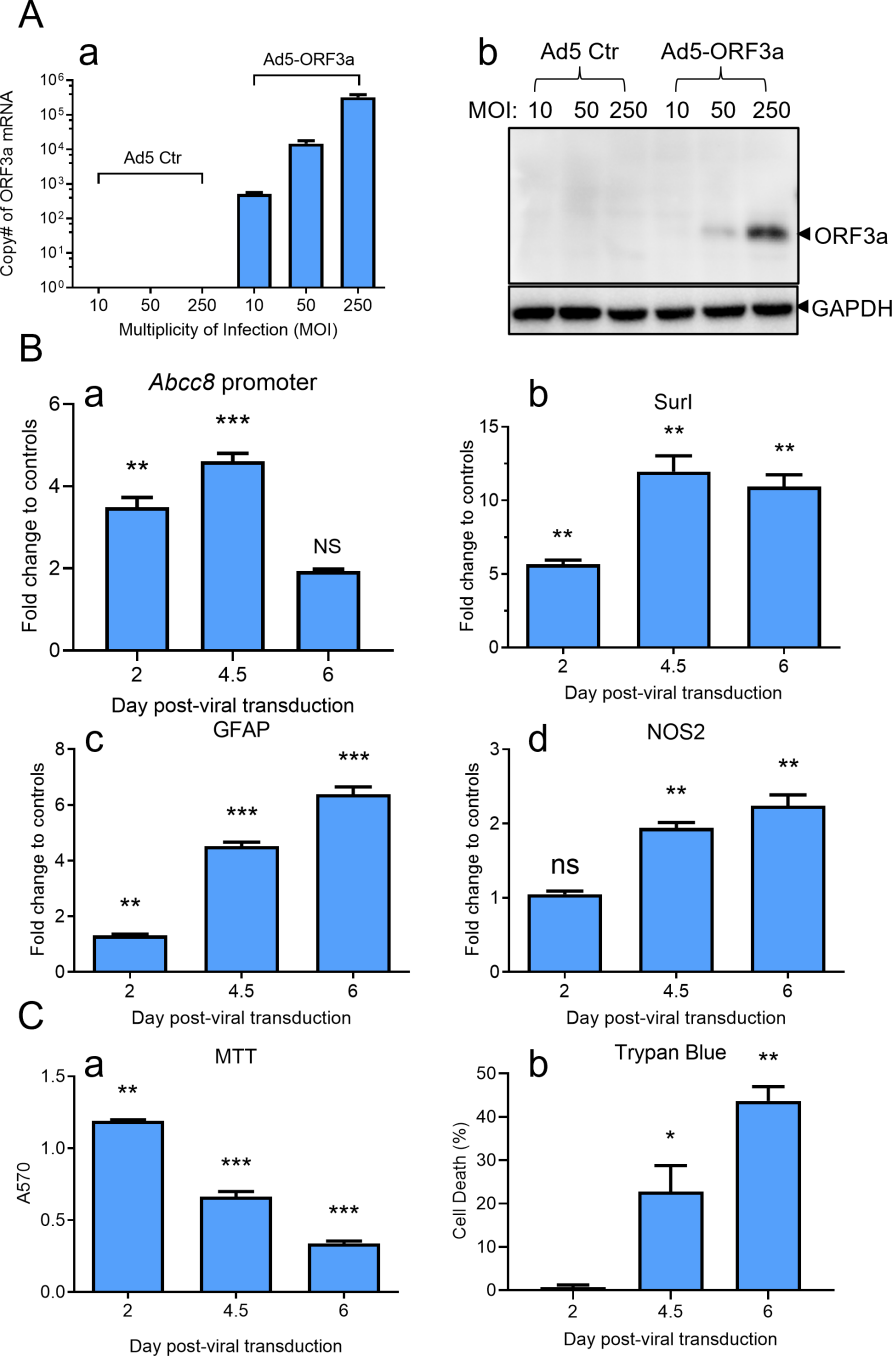
ORF3a expression induces the Sur1 elevation in the astrocytic cell line SNB19, leading to cell death over time. (**A**) Introduction of ORF3a protein via escalating multiplicities of infection (MOI) of ORF3a-producing adenovirus 5 (Ad5-ORF3a) in human astrocytic SNB19 cells results in a corresponding increase in ORF3a mRNA (a) and protein levels (b). Cells were transduced with the indicated MOIs of Ad5-ORF3a or control Ad5 (Ad5 Ctr) and collected at 5 days post-viral transduction (*Dpt*). (**B**) ORF3a expression activates the Abcc8/Sur1 promoter activity (a), leading to transcriptional upregulation of Sur1 (b), GFAP (c), and NOS2 (d), as measured by qRT-PCR in SNB19 cells. Cells were transduced with MOI 300 of Ad5-ORF3a or Ad5 Ctr and collected over a time period of 6 *Dpt*. Promoter activity was assessed using an Abcc8 promoter firefly luciferase assay (57). (**C**) ORF3a expression induces cell death in SNB19 cells, as determined by MTT assay (a) and trypan blue exclusion assay (b). Statistical significance is denoted as follows: * for *P*  <  0.05; ** for *P*  <  0.01; *** for *P*  <  0.001.

To assess whether ORF3a itself could drive the increase in Sur1, we developed an *ORF3a*-expressing adenovirus 5 (Ad5-ORF3a) system and investigated the impact of ORF3a in various astrocytic cell lines. In SNB19 cells, a human glioblastoma cell line derived from astrocytes, transducing Ad5-ORF3a at increasing multiplicities of infection (MOIs) resulted in a proportional increase in ORF3a mRNA levels by 5-day post-viral transduction (*dpt*) (Fig. 2A–a). The ORF3a protein became detectable at MOIs 50 and higher (Fig. 2A–b), while neither mRNA nor protein expression was detected in Ad5 empty vector controls, confirming an ORF3a-specific expression. Note that we used a hexon-based adenovirus titration assay to measure the titer of Ad5-ORF3a. This assay quantifies the hexon protein, a major structural component of the adenovirus capsid. However, it does not distinguish between infectious and non-infectious viral particles that contain hexon. As a result, the measured titer may overestimate the actual number of infectious particles capable of initiating a productive infection in SNB19 cells, which may explain the relatively high MOI required in these experiments.

We monitored Sur1 expression in SNB19 cells transduced by Ad5-ORF3a through examining the transcriptional activation of *Abcc8*/Sur1 over 6 days. Expression of ORF3a triggered activation of the *Abcc8*/Sur1 promoter activity, which was measured by an *Abcc8* promoter luciferase assay, and showed a peak of the promoter activity at 4.5 *dpt* (Fig. 2B–a) (57). Following the *Abcc8*/Sur1 promoter activation, the maximal transcriptional activity of Sur1, as measured by quantitative reverse transcription PCR (qRT-PCR), was achieved around 4.5 *dpt* and plateaued by 6 *dpt* (Fig. 2B–b). Alongside the ORF3a-induced Sur1 upregulation, GFAP, a marker of reactive astrocytes and brain injury (58, 59), and NOS2, a downstream Sur1 effector (60), also showed progressive increases within this timeframe (Fig. 2B–c–d). This upregulation coincided with a marked reduction in cell proliferation and viability, as indicated by MTT and trypan blue assays, accompanied by elevated cell death (Fig. 2C–a–b). The ORF3a-induced effects in SNB19 cells were consistent with our findings in other astrocyte cell lines, including human neuroblastoma SH-SY5Y (Fig. S2) and mouse neuroblast neuro-2a (N2a) cells (Fig. S3), demonstrating a conserved ORF3a impact on astrocyte-derived cell lines across species.

### NF-κB-dependent and -independent cytokine pathways in ORF3a-induced Sur1 expression and astrocyte cell death

**Fig 3:**
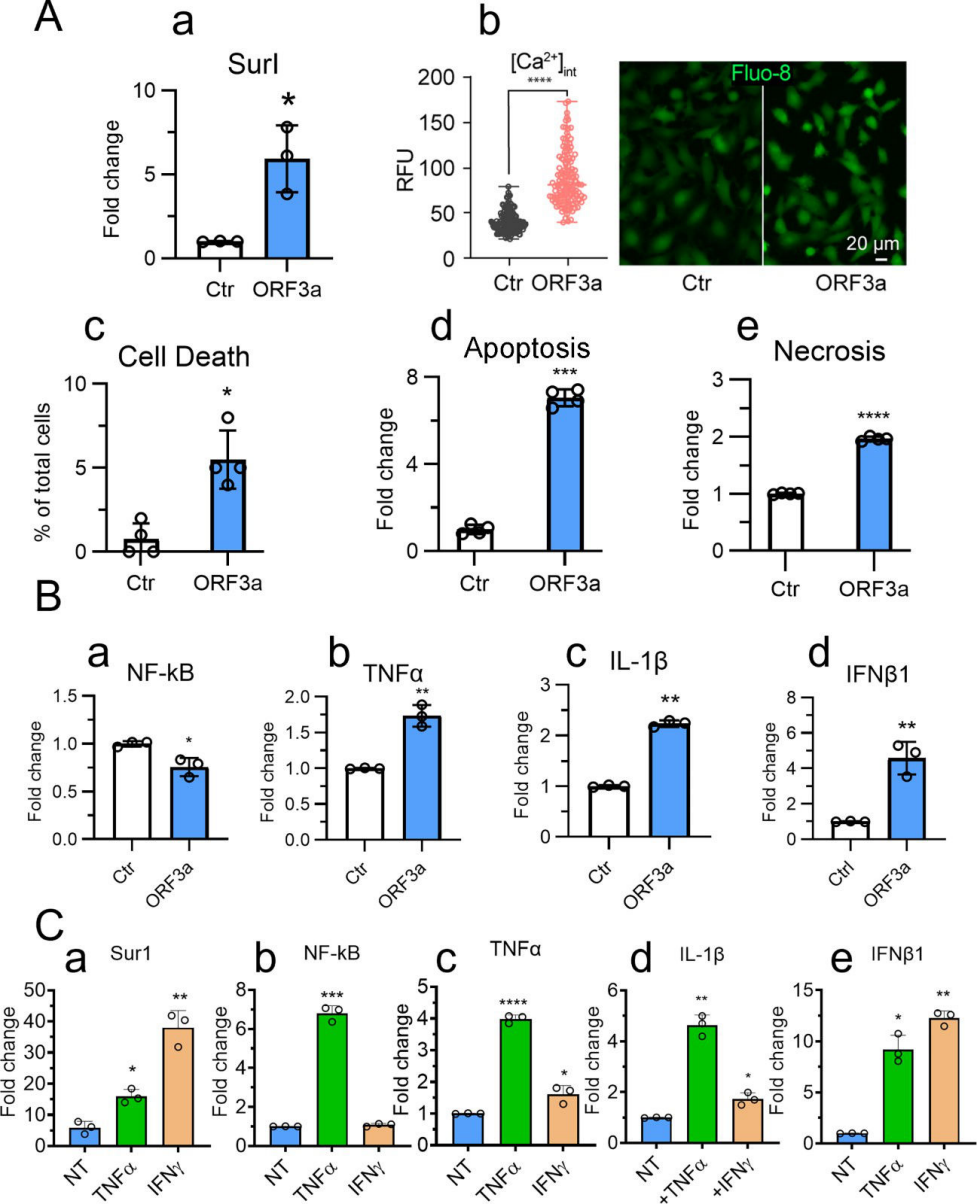
Inducible ORF3a expression leads to Sur1 activation, intracellular Ca²^+^ influx, and apoptotic astrocyte cell death through NF-κB-dependent and -independent cytokine signaling pathways. (**A**) Dox-inducible expression of ORF3a activates Sur1 (a), triggers intracellular Ca²^+^ influx (b), and induces apoptosis and necrosis in SNB19 astrocytic cells (c–e). (**B**) Inducible ORF3a expression shows no clear effect on NF-κB activation (a) but modestly increases cytokine levels, including TNFα, IL-1β, and IFNβ1 (b–d). (**C**) Treatment with TNFα or IFNγ enhances Sur1 activation in ORF3a-expressing cells (a). TNFα stimulates NF-κB activation (b), TNFα expression (c), and NF-κB-dependent cytokine production, such as IL-1β (d). IFNγ primarily drives IFNβ1 production (e). SNB19 astrocytic cells stably expressing ORF3a or EGFP (control) under a Dox-inducible promoter (pLVX-TetOne-Puro-ORF3a or -EGFP plasmid, 100 ng/mL dox) were used. Cells were collected on day 5 post-gene induction. TNFα or IFNγ (20 ng/mL) was added 24 h after dox induction, and cells were subsequently collected 24 h after. Immune marker levels were measured by qRT-PCR. Intracellular Ca²^+^ influx was assessed using the Fluo-8 Calcium Assay Kit (AAT Bioquest) 48 h post-Dox induction. The left graph in panel B represents the analysis of 153 cells using Mann-Whitney statistical testing. Statistical significance is denoted as follows: * for *P*  <  0.05; ** for *P*  <  0.01; *** for *P*  <  0.001.

We next investigated the molecular mechanisms underlying ORF3a-induced Sur1 elevation. To minimize potential artificial effects from ORF3a protein overproduction, we generated SNB19 cells that stably express ORF3a or an enhanced green fluorescent protein (EGFP) control using a doxycycline (Dox)-inducible pLVX-TetOne-Puro plasmid system. The addition of Dox (100 ng/mL) enabled inducible expression of ORF3a or EGFP, and cells were collected for analysis at 48 h post-induction (*hpi*). The inducible ORF3a expression in SNB19 cells resulted in a significant increase of Sur1 expression (Fig. 3A–a). Given that Sur1-regulated ion channels facilitate Ca²^+^ influx (35, 60), we evaluated ORF3a-induced Ca²^+^ influx through Ca²^+^ imaging with a Fluo-8 assay (61). ORF3a expression resulted in elevated intracellular Ca²^+^ levels (Fig. 3A–b), leading to cell death by apoptosis and necrosis (Fig. 3A–c–e).

While ORF3a expression alone had minimal activation effect on NF-κB and only modestly elevated cytokines, such as TNFα, IL-1β, and IFNβ1 (Fig. 3B), the addition of TNFα or IFNγ to ORF3a-producing cells mimicking secreted cytokines from targeted or immune cells further augmented Sur1 and cytokine levels (Fig. 3C). IFNγ induced a more pronounced upregulation of Sur1 than TNFα (Fig. 3C–a). Supporting the role of TNFα in NF-κB activation (62), TNFα significantly enhanced NF-κB levels in ORF3a-producing cells, while IFNγ did not (Fig. 3C–b). TNFα induced greater TNFα and IL-1β production than IFNγ (Fig. 3C–c–d), whereas IFNγ prompted a stronger IFNβ1 response indicative of a potential NF-κB-independent pathway (Fig. 3C–e). These data suggest that the ORF3a-mediated Sur1 upregulation is in synergy with secreted cytokines, such as TNFα and IFNγ, likely released by targeted and infiltrating immune cells, such as infected glial cells in SARS-CoV-2-infected brain tissues (63), exacerbating neuroinflammation through both NF-κB-dependent and -independent signaling pathways.

### Correlation of ORF3a and Sur1 with reactive astrocytes and neuroinflammation-related astrocytic apoptosis in SARS-CoV-2-infected K18-hACE2 transgenic mice

**Fig 4:**
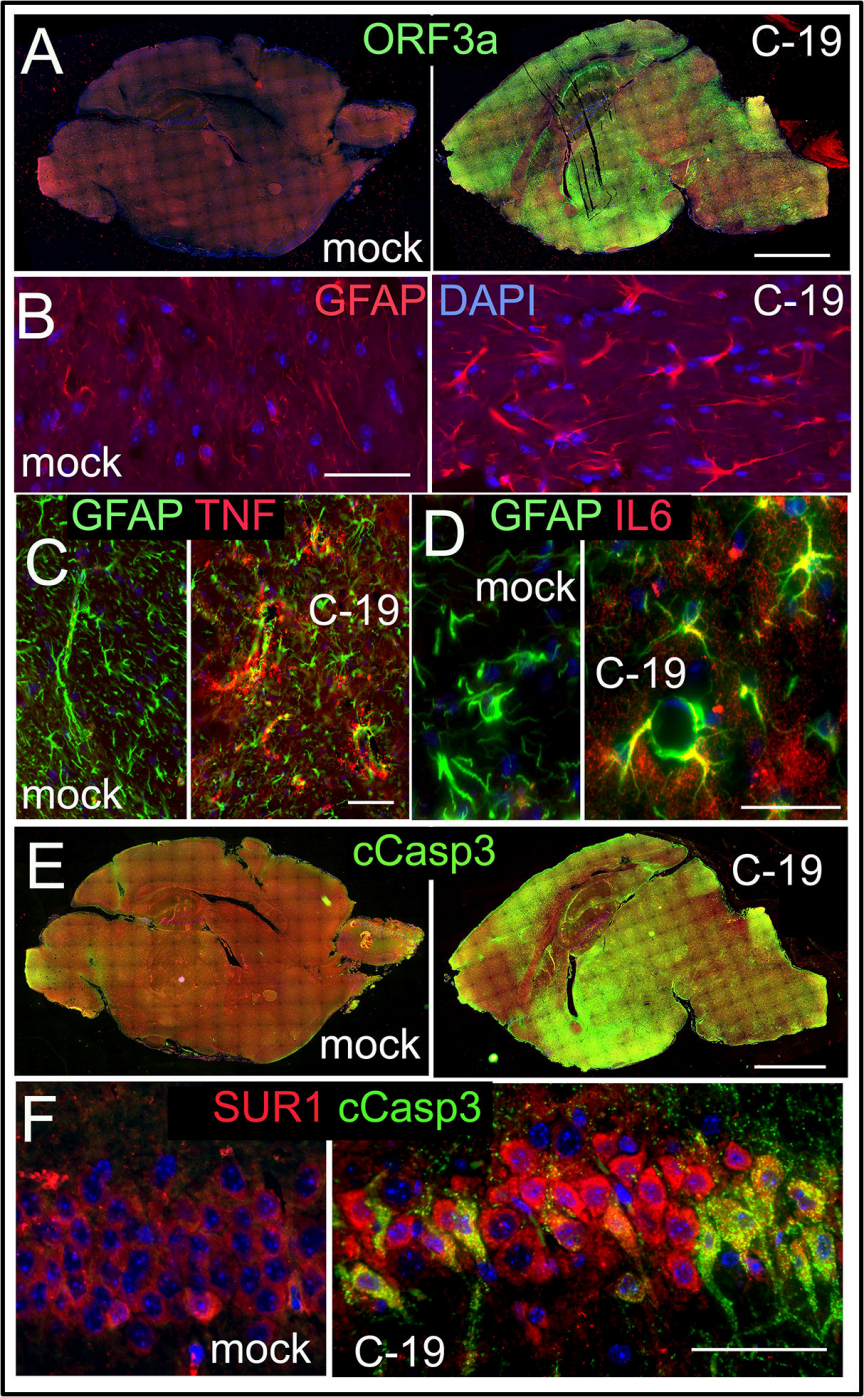
Correlation of the ORF3a expression with elevated Sur1 levels and neuroinflammation-associated astrocytic apoptosis in brain tissues of SARS-CoV-2-infected K18-hACE transgenic mice. (**A**) Immunostaining of ORF3a in brain cross-sections from mock-treated control (mock) mice (a, left) and SARS-CoV-2-infected (C-19) mice (a, right). Comparative immunostaining of GFAP between mock and C-19 (**B**). Co-immunolabeling of TNF (**C**) and IL-6 (**D**) in brain sections of mock Ctr and C-19 mice. Comparison of cleaved Caspase 3 (cCasp3) immunostaining of entire mouse brains between mock and C-19 (**E**) with Sur1 (**F**). Whole brains were collected at 8 *dpi* from K18-hACE transgenic mice infected with SARS-CoV-2 (2.5 × 10^4^ TCID₅₀). Scale bars in panels A and E are 1 mm; all others: 100 µm. Brain montages were stitched together using Nikon NIS-Elements imaging software.

To explore whether ORF3a expression correlates with neuroinflammation and astrocytic apoptosis in an animal model system, we employed K18-hACE2 transgenic (Tg) mice, which are susceptible to SARS-CoV-2 infection (23). Following infection with SARS-CoV-2, we collected brain tissues of both SARS-CoV-2-infected and mock-infected K18-hACE2 Tg mice at 8-days post-infection (*dpi*). Compared to mock controls, infected mice displayed widespread ORF3a expression throughout the brain (Fig. 4A). Consistent with the high levels of reactive astrocytes seen in the brain tissues of COVID-19 patients (Fig. 1), infected mice also showed elevated levels of reactive astrocytes compared to the mock controls (Fig. 4B). This increase was indicated by the higher expression of GFAP, a well-established marker for astrocyte reactivity and brain injury (58, 59). Additionally, proinflammatory cytokines TNF and IL-6 were observed within GFAP-positive astrocytes in infected tissues but were absent in mock controls, highlighting a strong inflammatory response (Fig. 4C through D). In parallel with the widespread ORF3a expression in infected mouse brains, there was extensive labeling of cleaved Caspase-3 (cCasp3; Fig. 4E), which marks apoptotic cell death in astrocytes. Sur1 was observed to co-reside with these cCasp3-positive astrocytes, establishing a link between ORF3a expression, Sur1 elevation, and astrocytic apoptosis (Fig. 4F). Collectively, these findings suggest that ORF3a contributes to neuroinflammation and astrocyte death in the infected mouse brain, reflecting similar neuropathological patterns seen in human COVID-19 cases (Fig. 1).

### Both wild-type (WT) and Omicron-associated T223I mutant ORF3a proteins induce Sur1-associated Ca^2+^ influx in human astrocytic cells

As an error-prone RNA virus (64), SARS-CoV-2 continues to mutate, and certain fixed ORF3a mutations are present in natural SARS-CoV-2 variants, especially those defined as variants of interest (VOI) or variants of concern (VOC) by the World Health Organization (WHO) that are often associated with higher mortality (25, 65, 66) and severe disease outcomes (65, 67–69). To examine whether the observed ORF3a effects are also present in those emerging ORF3a mutants, we extended our study to include the unique ORF3a T223I mutant associated with Omicron variants (21, 25, 70). The ORF3a T223I mutant was selected because some of the ongoing circulating viral variants are Omicron-derived viruses, which are in many ways different from the original WT virus both in viral pathogenicity and transmission (71). Additionally, our previous mutagenesis studies identified two distinct types of lysosome-associated (L-ORF3a, such as WT) and ER-associated (E-ORF3a like T223I) ORF3a proteins based on subcellular localization and cytotoxic effects (22, 72).

**Fig 5:**
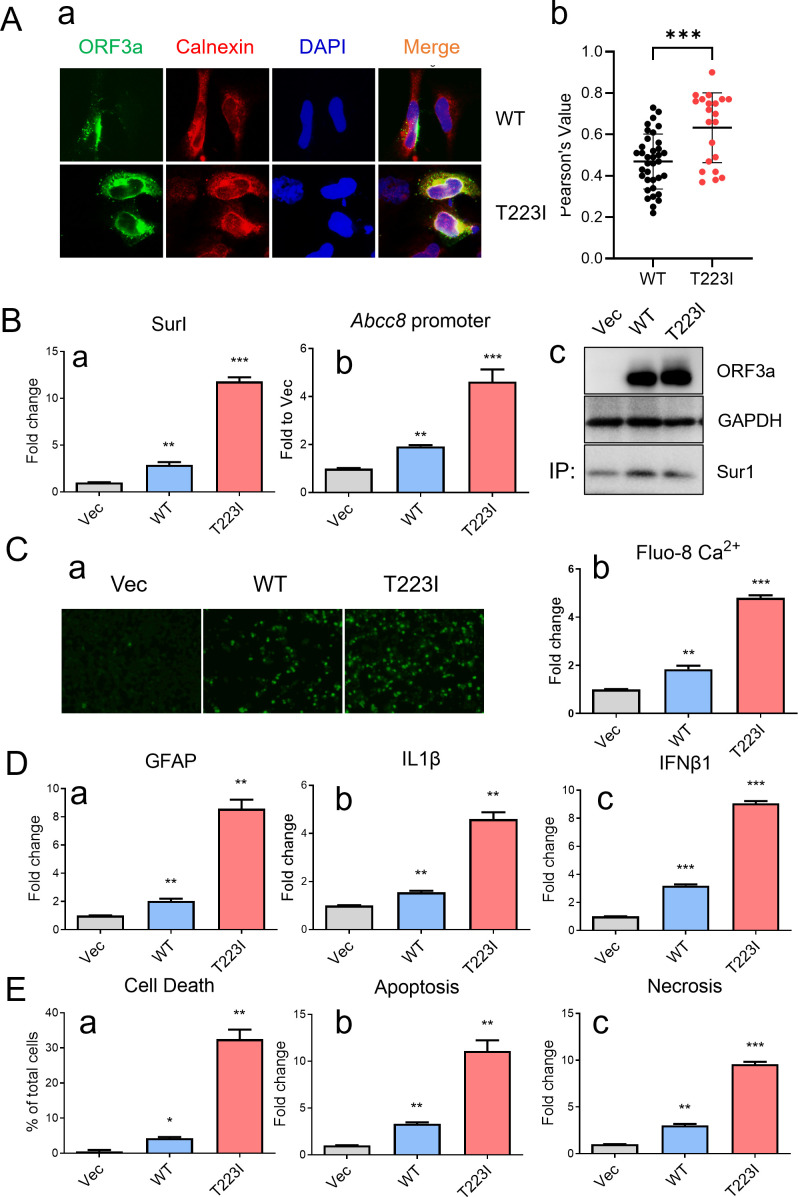
WT and Omicron-associated T223I mutant ORF3a induce Sur1-associated Ca²^+^ influx and apoptotic cell death in SNB19 cells via proinflammatory cytokine production. (**A**) Co-localization of WT and T223I mutant ORF3a with Calnexin, an ER membrane protein, was assessed at 24 *hpt* using IFA with an anti-Calnexin antibody (a) and quantified by calculating Pearson’s correlation coefficients and *P* values using Fiji (ImageJ2 with JACoP plugin) (21, 22, 72) and represented in a graph generated by Mann-Whitney analysis (b). A total of 20 and 14 random images were analyzed for WT and T223I, respectively, with the results shown as the mean and standard deviation of *P* values. (**B**) WT and T223I ORF3a induce transcriptional upregulation of Sur1 (a) and activate the Abcc8/Sur1 promoter activity (b). Protein expression of ORF3a and Sur1 was verified by western blot analyses. Sur1 detection was enhanced by immunoprecipitation prior to western blot analysis (c). (**C**) Elevated Sur1 expression correlates with intracellular Ca²^+^ influx induced by ORF3a, as visualized by Ca²^+^ imaging analysis (a) and quantified (b). Intracellular Ca²^+^ levels were measured using the Fluo-8 Calcium Assay Kit (AAT Bioquest) at 24 *hpt*. (**D**) Time-course experiments reveal concurrent elevation of GFAP (a), IL-1β (b), and IFNβ1 (c) following expressions of WT and T223I ORF3a. (**E**) Both WT and T223I ORF3a induce apoptosis and necrosis, as measured by the RealTime-Glo apoptosis and necrosis assay (Promega). SNB19 cells were transfected with pCAG plasmids encoding WT or T223I ORF3a. Cells were collected at 5 days post-transfection unless otherwise noted. All markers were measured by qRT-PCR. Statistical significance is indicated as follows: * for *P* < 0.05, ** for *P* < 0.01, and *** for *P* < 0.001.

To examine whether subcellular location of WT and T223I mutant ORF3a proteins remains similar in SNB19 cells to that in other human cells as we reported previously (21), we measured their presence in the ER compartment in SNB19 cells using the immunofluorescence assay. As expected, there was a significantly (*P* < 0.007) higher percentage of T223I mutant ORF3a protein residing in the ER with a median of Pearson’s correlation coefficient of 0.70 compared to WT ORF3a with a median of Pearson’s correlation coefficient of 0.48 (Fig. 5A). These observations are similar to what we observed in other human cells (21, 22, 72).

To examine whether the T223I mutant also induces Sur1 upregulation, we transfected SNB19 cells with plasmids expressing WT ORF3a, T223I mutant ORF3a, or an empty control vector. qRT-PCR analysis confirmed that both WT and T223I ORF3a proteins significantly elevated the mRNA level of Sur1 (Fig. 5B–a). Promoter activity assays for *Accb8*/Sur1 demonstrated that the T223I mutant elicited a higher level of *Accb8*/Sur1 expression than WT ORF3a (Fig. 5B–b). Western blot analysis further confirmed the production of WT and T223I ORF3a proteins with elevated Sur1 expression (Fig. 5B–c). In alignment with the observed Sur1 elevation, both WT and T223I ORF3a induced proportional increases in intracellular Ca^2+^ measured by Fluo-8 imaging (Fig. 5C–a–b). Additional qRT-PCR analysis showed corresponding transcriptional profiles of GFAP, IL-1β, and IFNβ1 (Fig. 5D), which corresponded to subsequent apoptosis and necrosis of SNB19 cells (Fig. 5E).

### Suppression of ORF3a-induced Sur1 expression and apoptotic cell death by glibenclamide and glycyrrhizin

**Fig 6:**
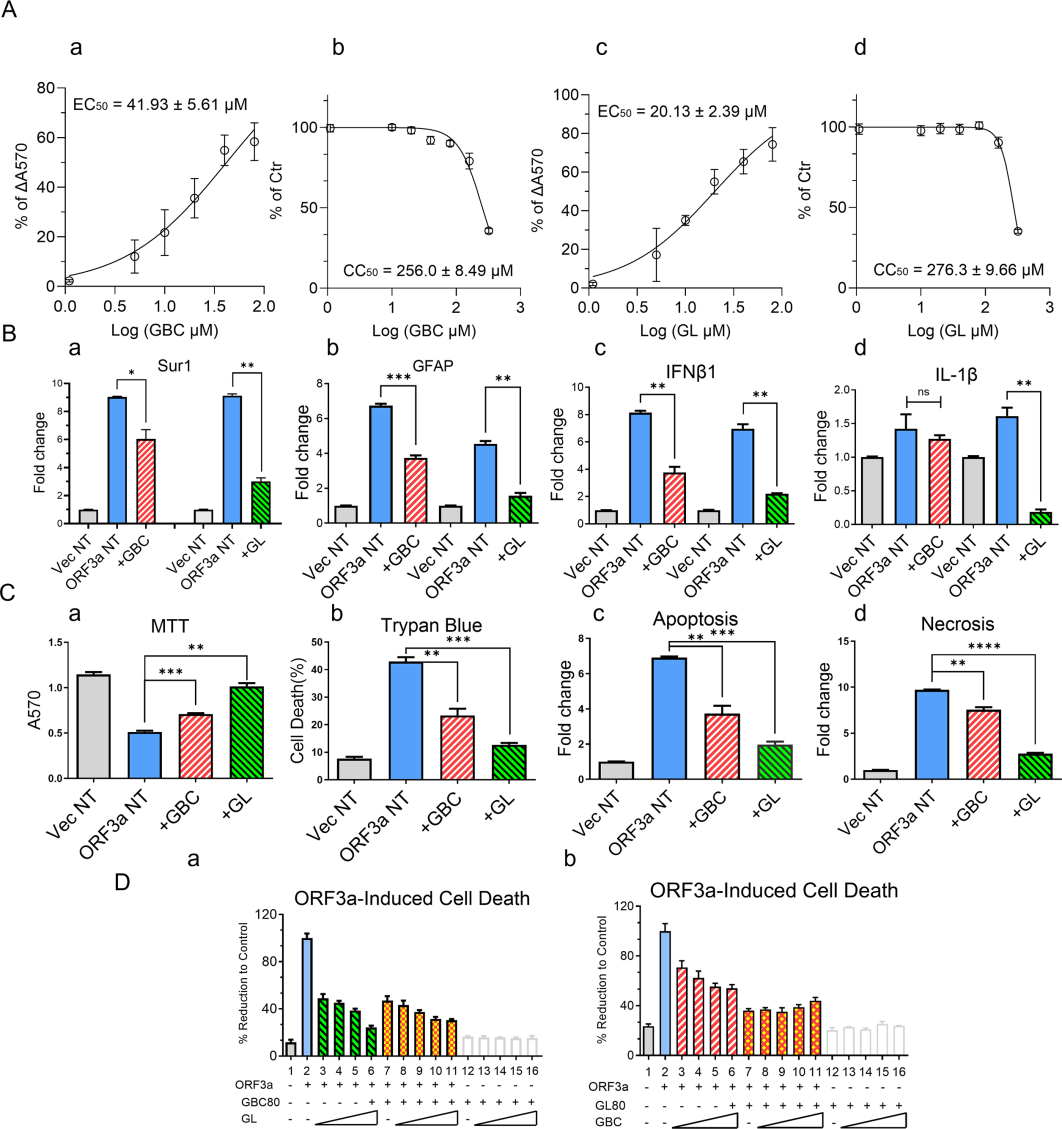
Glibenclamide (GBC) and glycyrrhizin (GL) suppress ORF3a-induced Sur1 elevation and apoptotic cell death in SNB19 cells. (**A**) Therapeutic potential of GBC and GL in suppressing ORF3a-induced cell death was assessed by determining the EC₅₀ and CC₅₀ over a range of seven doses (5.0–320.0 µM). The therapeutic index was calculated as the ratio of EC₅₀ to CC₅₀ based on MTT assay results. (**B**) Effects of GBC (left columns) and GL (right columns) on transcriptional profiles of *Sur1* (a), *GFAP* (b), and representative cytokines *IL-1β* (c) and *IFNβ1* (d). (**C**) GBC and GL mitigate ORF3a-induced apoptotic cell death, as measured by the MTT assay (a), trypan blue exclusion assay (b), and RealTime-Glo apoptosis (c) and necrosis (d) assays. (**D**) Combined treatment with GBC and GL exhibits an additive inhibitory effect on ORF3a-induced cell death in SNB19 cells. SNB19 cells were transduced with Ad5-ORF3a (dashed bars) or Ad5 control (white open bars) and treated with increasing doses of GBC (a) or GL (b) ranging from 20 to 80 µM. Treatments were applied to Ad5-ORF3a-expressing cells either alone or in combination with the other drug (80 µM), as well as to Ad5-transduced control cells lacking ORF3a expression. Measurements were performed at day 5 post-transduction. Transcriptional expression of *Sur1*, *GFAP*, *IL-1β*, and *IFNβ1* was analyzed by qRT-PCR. Statistical significance is denoted as follows: * for *P*  <  0.05; ** for *P*  <  0.01; *** for *P*  <  0.001.

To explore potential strategies for blocking Sur1-mediated neuroinflammation and neurotoxicity induced by ORF3a, we tested the inhibitory effects of glibenclamide (GBC, aka glyburide), an FDA-approved antidiabetic drug that specifically targets Sur1 channels (42). A seven-dose range of GBC from 5.0 to 320.0 µM was employed to determine its half-maximal effective concentration (EC_50_) against ORF3a-induced cell death in SNB19 cells using the MTT assay. GBC had an EC_50_ of 41.93 ± 5.61 µM, a 50% cytotoxic concentration (CC_50_) of 256.0 ± 8.49 µM, and a therapeutic index (TI) of 6.11 ± 0.81 (Fig. 6A–a–b). Additionally, our previous studies identified glycyrrhizin (GL), a natural compound, as an inhibitor of ORF3a effects in renal cells (21). In this study, we further evaluated the therapeutic potential of GL in SNB19 cells. The MTT analysis revealed that GL exhibited an EC_50_ of 20.13 ± 2.39 µM, a CC_50_ of 276.3 ± 9.66 µM, and a TI of 13.73 ± 1.44 (Fig. 6A–c–d).

Given the comparable EC_50_ values of GBC and GL, their inhibitory effects were tested at equal concentrations (80 µM) against ORF3a-induced Sur1 and GFAP expressions, cytokine production, and apoptotic cell death in SNB19 cells. As the control, ORF3a expression in SNB19 cells increased Sur1 levels by approximately ninefold. Treatment with GBC reduced Sur1 levels by 33.1% (Fig. 6B–a, left), while doubling the concentration of GBC further decreased Sur1 expression to a 50.8% reduction, indicating a dose-dependent response (Fig. S4A). Treatment with GL reduced Sur1 levels by 67.0% (Fig. 6B–a, right). GL also showed stronger inhibitory effects on GFAP and IFNβ1 expressions compared to GBC (Fig. 6B–b–d). Interestingly, while GBC treatment did not affect IL-1β levels, GL significantly downregulated IL-1β, though the ORF3a-induced increase in IL-1β was marginal (Fig. 6B–d). However, the differential effects of GBC and GL on IL-1β were reproducible (Fig. S4D). Regarding ORF3a-induced cell death, GL also demonstrated a stronger reduction effect compared to GBC (Fig. 6C).

To assess the combined inhibitory effects of GBC and GL, we carried out a reciprocal dosing experiment, where one drug’s concentration was fixed at 80 µM, while the other was varied incrementally from 20 to 80 µM. Both GBC and GL independently reduced ORF3a-induced cell death in a dose-dependent manner. However, the combination of GBC and GL resulted in a small but significant additional reduction in ORF3a-induced cell death compared to GBC or GL alone (Fig. 6D). These findings suggest an additive effect of GBC and GL on the suppression of ORF3a-induced cell death.

## DISCUSSION

In this study, we identified a functional link between SARS-CoV-2 infection, the expression of the viral protein ORF3a, and COVID-19-associated neuroinflammation, along with apoptosis and necrosis of astrocytes in postmortem brain tissues from individuals with COVID-19 (Fig. 1). Specifically, the presence of ORF3a was associated with astrocyte activation characterized by an upregulation of the ion channel proteins Sur1 and Trpm4, which contribute to the activation of proinflammatory cytokines and subsequent apoptotic cell death. Comparison of both low-resolution and high-magnification images (Fig. 1; Fig. S1) with previously published studies reveals generally similar patterns of antigen staining (6, 73, 74). However, some subtle differences are observed, including variations in staining morphology and the relatively higher intensity seen in our images. To our knowledge, this is the first study to report immunostaining of ORF3a protein in postmortem brain tissues. Therefore, in addition to possible technical differences, the distinct staining characteristics may reflect the unique membrane-bound nature of the ORF3a protein (25). Our *in vitro* experiments further demonstrated that ORF3a expression alone is sufficient to induce the elevation of the Sur1 ion channel, intracellular Ca^2+^ influx, and GFAP, a marker of reactive astrocytes and brain injury (58, 59), ultimately leading to the induction of proinflammatory cytokine production and apoptotic cell death in the astrocytic cell line SNB19 (Fig. 2). Mechanistically, we demonstrated that ORF3a-induced neuroinflammation is mediated by Sur1-regulated ion channels, leading to increased Ca²^+^ influx and subsequent apoptotic astrocyte death, which is consistent with the known functions of Sur1-regulated ion channels (35). Additionally, we found that ORF3a-induced Sur1 expression occurs via NF-κB-mediated cytokine production, specifically TNFα and IL-1β, as well as through an NF-κB-independent pathway involving IFNβ1 of the IFNγ-mediated pathway (Fig. 3). Notably, the effects of ORF3a were further amplified by the addition of cytokines, such as TNFα and IFNγ. This augmentation is likely due to the release of TNFα and IFNγ either directly from ORF3a-expressing astrocytes, which serve as immune effector cells, or from other infiltrating immune cells, such as microglia, in SARS-CoV-2-infected brain tissues (Fig. S1) (63). However, it remains unclear whether this cytokine release is astrocyte-specific or due to contributions from other immune cells, requiring further investigation. Given that astrocytes play an active role in innate immunity by regulating inflammation (75), it is plausible that ORF3a expression not only triggers the production and secretion of TNFα and IFNγ but also interacts with other immune cells within the brain microenvironment. Regardless of the TNFα and IFNγ source, co-stimulation with these cytokines significantly exacerbated the effects of ORF3a, while the same TNFα and IFNγ treatments to control cells lacking ORF3a expression showed no such response (Fig. 3C), indicating an ORF3a-specific stimulatory effect.

Using a K18-hACE2 transgenic mouse model (23), we demonstrated a link between SARS-CoV-2 infection, ORF3a expression, Sur1-associated neuroinflammation, and apoptotic astrocyte death, similar to what we observed in postmortem brain tissues of individuals with COVID-19 (Fig. 1), thereby relating ORF3a to SARS-CoV-2-induced brain injury (Fig. 4). Our findings in the K18-hACE2 mice aligned with an earlier study in which ORF3a introduction into the mouse brain via an AAV9 vector delivery system led to the rapid onset of neuroinflammation, neurodegeneration, and neurological impairment (17). In that report, it shows that ORF3a expression disrupts the autophagy-lysosomal pathway, resulting in the neuronal accumulation of α-synuclein and glycosphingolipids, which are associated with neurodegenerative diseases, such as Parkinson’s disease and frontotemporal dementia (76–78). This suggests that ORF3a-induced aggregation of α-synuclein and glycosphingolipids may contribute to the pathogenesis of these neurodegenerative disorders. Indeed, elevated levels of glycosphingolipids have been linked to frontotemporal dementia (76), while intracellular α-synuclein aggregates, a hallmark of Parkinson’s disease, have been detected in the brains of SARS-CoV-2-infected individuals and macaques (79, 80). Thus, these findings support the assertion that ORF3a expression may drive neuropathogenesis by disrupting the autophagy-lysosomal pathway. Note that while the K18-hACE2 transgenic mouse model has been widely used to study SARS-CoV-2 infection, it carries several limitations relevant to our study. The ectopic expression of human ACE2 results in a non-physiological overexpression of ACE2, particularly in neural cells (81). This leads to a pattern of neuroinvasion and brain pathology that is more severe than typically seen in humans and not representative of what is observed in other animal models, including WT mice, hamsters, and non-human primates. Consequently, the extent of neuroinflammation and astrocytic injury observed in K18-hACE2 mice may be amplified compared to natural infections in humans. Despite these limitations, the K18-hACE2 model remains a valuable tool for initial mechanistic studies of SARS-CoV-2 pathogenesis, particularly when evaluating the effects of specific viral proteins, such as ORF3a. To address these concerns and strengthen the translational relevance of our findings, our future studies will incorporate mouse-adapted SARS-CoV-2 strains, such as MA10 or MA30 in WT C57BL/6 mice (82, 83). These models are believed to better mimic the natural course of SARS-CoV-2 infection in humans without the confounding effect of artificial ACE2 overexpression.

The impact of ORF3a on autophagy and lysosomal function is well documented and extensively reviewed (84). When ORF3a localizes to the lysosomal membrane of infected cells, it triggers a host cellular antiviral autophagy response aimed at lysosomal viral clearance (85). This activation involves the HOPS complex, which promotes the fusion of autophagosomes with lysosomes (85, 86). However, ORF3a counteracts this antiviral autophagic response by disrupting the fusion of lysosomes with autophagosomes through its direct interaction with VPS39, a key component of the HOPS complex (22, 85, 87). As a result, blockade of the host’s antiviral autophagy response by ORF3a enhances viral replication and increases lysosomal exocytosis-mediated viral egress (88). Additionally, ORF3a disrupts host lipid homeostasis, leading to the accumulation of lipid droplets (70), which may contribute to the buildup of α-synuclein and glycosphingolipids, further promoting viral production (17, 89). Notably, the Omicron-associated ORF3a T223I mutant impairs the interaction between ORF3a and VPS39, as well as lipid droplet accumulation, resulting in reduced viral replication. This may partly explain the attenuated pathogenicity of Omicron strains (70).

Through mutagenesis studies, we previously demonstrated that the T223I mutant protein represents one of two distinct types of ORF3a proteins with differences in subcellular localization (21, 22, 72). Unlike WT ORF3a, the T223I mutant protein predominantly localizes to the ER membrane rather than the lysosomal membrane (Fig. 5A) (21, 22) potentially due to reduced efficiency in intracellular transport from the ER to lysosomes (72). This unique characteristic of the T223I mutant suggests it may exert distinct effects on the Sur1 ion channel and its associated cytotoxicity in neural cells. Consistent with our earlier findings that ER-associated ORF3a variants generally induce greater cytopathic effects compared to those associated with lysosomal membranes, the T223I mutant protein production indeed resulted in more pronounced cell death than the WT ORF3a in several astrocytic cell lines, including SNB19, SH-SY-5Y, and N2a cells (Fig. 5E; Fig. S2 to S4). This heightened cytotoxicity is likely due to increased levels of Sur1-regulated channels, elevated intracellular Ca²^+^ influx, and a stronger proinflammatory response compared to WT ORF3a (Fig. 5B through D).

ORF3a is a viral transmembrane protein that functions presumably as a viroporin, disrupting host cellular ion homeostasis (25, 90, 91). An earlier study using a liposome system demonstrated that ORF3a forms a nonselective, Ca²^+^-permeable cation channel (26). Structural analysis further revealed that ORF3a directly binds to Ca²^+^ (92), suggesting that it may influence host cellular ion homeostasis through ion channel-mediated Ca²^+^ regulation. However, another study suggests that ORF3a is not an ion channel protein (93). Nonetheless, the precise mechanisms by which ORF3a regulates host cellular ion channels remain unclear. In this study, we present, for the first time, evidence that ORF3a promotes the activation of Sur1-regulated cation channels, thereby disrupting host cellular ion homeostasis. Our findings indicate that the activation of Sur1-regulated ion channels contributes to ORF3a-induced neuroinflammation and neurotoxicity in astrocytes, as evidenced by the significant elevation of GFAP, a marker of reactive astrocytes and brain injury (58, 59).

Sur1-mediated ion channels are not constitutively expressed in astrocytes (34). Instead, their expression is upregulated in response to various neuroinflammatory diseases, brain injuries, and pathological brain conditions, including TBI, SAH, MS/EAE, and HIV-1 infection (34, 37–41, 94). Therefore, the expression of Sur1 observed in ORF3a-expressing cells likely represents a specific cellular response to ORF3a during SARS-CoV-2 infection. However, the precise molecular mechanisms by which ORF3a induces Sur1 ion channel activation remain unclear. Previous studies suggest that ORF3a may function as a pore-forming protein influencing Ca²^+^ channels (26, 91). While we cannot exclude this possibility, our observations indicate an alternative scenario where ORF3a may interact with Sur1, either directly or indirectly, to form a novel channel that impacts Ca²^+^ influx. Supporting this hypothesis, ORF3a has been shown to bind to the chloride ion channel protein CLCC1, which is present in a SARS-CoV-2-infected brain tissue (95, 96). Interestingly, CLCC1 primarily localizes to the ER membrane, and its interaction with ORF3a induces ER stress responses (96). Additionally, CLCC1 plays a role in regulating ER Ca²^+^ homeostasis, with disruptions to this channel leading to neurodegeneration (97). Given that Sur1 is expressed on both the plasma membrane and the ER (34, 98), where both WT and T223I ORF3a are known to exert their effects on Sur1-regulated ion channels and Ca²^+^ homeostasis, it would be of interest to investigate whether Sur1 interacts with these two types of ORF3a proteins in a membrane-specific manner. Exploring these interactions may shed light on the differential effects of WT and T223I ORF3a on cellular Ca²^+^ dynamics and associated neurotoxicity.

Since ORF3a upregulates Sur1, leading to apoptosis and necrosis in astrocytes, it is not surprising that GBC, a Sur1-specific inhibitor, effectively suppresses ORF3a-induced apoptosis and necrosis (Fig. 6). Our previous studies have shown that GBC directly binds to Sur1, inhibiting the opening of the Sur1-Trpm4 channels (34, 99), which depolarizes the membranes of ischemic or traumatized brain cells (100, 101). GBC has shown promising results in preclinical and clinical studies for treating various neuroinflammatory conditions associated with Sur1-regulated channels and brain injuries. The pharmacological inhibition of Sur1 by GBC has led to significant improvements in clinical outcomes for neurocognitive disorders (40, 42–45).

In this study, we demonstrated that GBC inhibits Sur1-associated neuroinflammation and neurotoxicity induced by ORF3a, with a therapeutic index of 29.7. While this therapeutic index is considered moderate for drug development, it is important to note that in cell culture systems, the majority of GBC binds to fetal bovine serum (FBS) (60, 102, 103). Consequently, *in vitro* EC_50_ values significantly underestimate the actual *in vivo* EC_50_ of GBC, which is approximately 48 nM at the physiological human body pH of 7.4, exhibiting high affinity and specificity for Sur1 (60, 102, 103). Therefore, despite a moderate therapeutic index shown *in vitro*, our findings underscore that GBC is highly likely to be an exceptionally potent Sur1 inhibitor *in vivo* against the ORF3a effects, as demonstrated in our previous clinical studies and trials (40, 42–45, 104, 105).

In addition to GBC, we further demonstrated that GL effectively suppresses the ORF3a-mediated upregulation of Sur1 channels, GFAP expression, and proinflammatory cytokine production (Fig. 6B, right), leading to a significant reduction in apoptosis and necrosis in SNB19 cells (Fig. 6C, right). GL is a well-known natural compound recognized for its anti-inflammatory and anti-viral properties against various viral infections, including SARS-CoV-2 (46–48). Mechanistically, GL inhibits HMGB1 (High Mobility Group Box 1), a damage-associated molecular pattern (DAMP) protein that is released in response to cellular stress or tissue damage (106, 107). In our recent study on ORF3a’s role in COVID-associated kidney injury, we demonstrated that ORF3a triggers HMGB1 activation, evidenced by its nuclear release following ORF3a expression (21). Additionally, GL was shown to suppress ORF3a-induced apoptosis and necrosis in renal cells while also inhibiting viral replication in kidney epithelial cells (21). Given that the focus of this study was on the effect of ORF3a on Sur1 channels, we did not specifically measure HMGB1 activity in the context of astrocytes. However, we hypothesized that HMGB1 may behave similarly in these cells as it does in renal cells (21, 108, 109). Notably, our previous findings indicate that ORF3a interacts with HMGB1 (21), and prior research has shown that GL binds directly to HMGB1 (110). Thus, it would be of interest in future studies to examine whether GL disrupts the ORF3a-HMGB1 interaction to mitigate ORF3a’s effects in astrocytes.

Another important issue we have not yet addressed in this study is whether GBC and GL can cross the BBB, as drug delivery to the brain remains a major challenge due to the restrictive nature of the BBB. Several lines of evidence from our studies and others suggest that ORF3a and these two compounds may influence and/or penetrate the BBB under certain conditions. For instance, our earlier work demonstrated that GBC can cross a compromised BBB and accumulate in regions associated with neurological lesions (34, 43). In addition, SARS-CoV-2 infection is known to disrupt BBB tight junctions (111), and ORF3a has been shown to induce astrocytic cell death (Fig. 4E and F) and disrupt perivascular astrocytic endfeet (Fig. 1B–a), which play a crucial role in maintaining BBB integrity (55). These disruptions may facilitate GBC entry into the brain. As for GL, its major metabolite, 18β-glycyrrhetinic acid (18β-GL), has been shown to cross the BBB in rats (112). That said, there is currently no direct evidence demonstrating that ORF3a alters BBB permeability or that pharmacologically active concentrations of GBC or GL reach ORF3a-affected brain regions following systemic administration. This critical question remains to be addressed in our future studies.

Interestingly, the combined use of GBC and GL suggests an additive effect in suppressing ORF3a-induced cell death (Fig. 6D). These results indicate that their inhibitory effects on ORF3a might be interconnected through a shared mechanistic pathway. However, there is currently no direct evidence linking Sur1 and HMGB1 function, particularly in the context of neuroinflammation and brain injury. Nevertheless, both Sur1 and HMGB1 are known to be upregulated in injured brain cells, contributing to neuroinflammation and subsequent brain injury (113, 114). It is, therefore, conceivable that the activation of Sur1-regulated channels could lead to HMGB1 activation via downstream effects, such as increased intracellular Ca^2+^ levels triggered by Sur1 channel opening (Fig. 3A–B; Fig. 5C). These elevated Ca^2+^ levels may then initiate cellular stress responses, including the activation of HMGB1 (60). Thus, Sur1 and HMGB1 may be functionally linked through their respective roles in neuroinflammatory responses and cellular injury, potentially contributing to the pathological outcomes associated with ORF3a-induced ion dysregulation, neuroinflammation, and neurocytotoxicity.

A limitation of this study is that many of the mechanistic analyses were performed using immortalized astrocytic and neuronal cell lines, which may not fully recapitulate the physiological behavior of primary human or murine astrocytes. Although these models offer important insights, validation in primary cells and *in vivo* systems is critical for confirming relevance to human disease. Notably, our findings showed elevated Sur1 expression in the brains of SARS-CoV-2-infected K18-hACE2 mice, supporting *in vivo* relevance. Our future studies will include experiments using primary human and mouse astrocytes, as well as animal models, such as MA10 or MA30 mice (82, 83). These efforts aim to confirm the role of ORF3a in mediating astrocyte death and neuroinflammation and test the therapeutic potential of glibenclamide in mitigating these effects.

In summary, we identified ORF3a as a key viral factor contributing to COVID-related neuroinflammation and neurotoxicity in the infected brain during SARS-CoV-2 infection. This causal relationship between ORF3a and COVID-related neuroinflammation was initially observed in postmortem brain tissues of COVID-19 patients and subsequently validated *in vitro* through independent ORF3a expression. A similar association was also found in brain tissues of SARS-CoV-2-infected K18-ACE2 transgenic mice. Mechanistically, we demonstrated that the neuroinflammatory effects of ORF3a are likely to be mediated by the activation of astrocytes and Sur1-regulated ion channels, which trigger the production of proinflammatory cytokines and lead to apoptotic cell death of astrocytes. Furthermore, we discovered that the FDA-approved drug GBC and the natural compound GL effectively counteract the neuroinflammatory effects of ORF3a. These findings highlight a novel clinical and functional link between ORF3a and COVID-associated neuroinflammation and neurotoxicity, suggesting that repurposing GBC along with GL could potentially offer a promising therapeutic strategy to mitigate COVID-associated neuroinflammation and neurotoxicity and prevent COVID-19-associated neurocognitive complications.

## MATERIALS AND METHODS

### Cells and growth media

SNB19 (RRID:CVCL_0535 from National Cancer Institute) is a human glioblastoma cell line that develops from astrocytes. It was maintained in RPMI 1640 medium supplemented with L-glutamine and 10% FBS (Quality Biological/Fisher Scientific Cat#: 112025101) and 100 units/mL penicillin plus 100 µg/mL streptomycin (41, 115). The SNB19 cell line was also used to establish in this study cell clones that stably produce ORF3a or EGFP by using a DoX-inducible pLVX-TetOne-Puro plasmid system (Takara Bio). Dox-inducible production of ORF3a or EGFP (as a control) proteins was achieved by adding Dox at a concentration of 100 ng/mL, and cells were collected at 24–48 h post-Dox induction. Human neuroblastoma SH-SY5Y cells were cultured in a medium composed of a 1:1 mixture of Eagle’s minimal essential medium (EMEM) and F12K. Mouse N2a cells were cultured in Dulbecco’s modified Eagle’s medium (DMEM) supplemented with 10% heat-inactivated FBS, 0.3% glutamine, and antibiotics (100 U/mL penicillin, 100 µg/mL streptomycin) at 37°C and 5% CO_2_ (116, 117).

### ORF3a expression and delivery systems

We employed three different approaches to express ORF3a in cells. First, an ORF3a-expressing Ad5-ORF3a construct was generated using the Adeno-X Adenoviral System 3 (Takara Bio) following the manufacturer’s instructions, with correct insertion of the ORF3a nucleotide sequence confirmed via restriction digestion and Sanger sequencing and viral titers quantified using the hexon-based adenovirus titration kit (Takara Bio). Second, a Dox-inducible ORF3a expression system in SNB19 cells was established and used to achieve stable, low-level expression of ORF3a, minimizing potential artifacts from protein overproduction. Third, WT and T223I mutant ORF3a constructs were expressed using pCAG plasmids as we previously described (21, 72). For transfections, SNB19 and N2a cells were transfected with pCAG-ORF3a plasmids using Invitrogen lipofectamine stem transfection reagent (Thermo Fisher, Cat# STEM00008), while SH-SY5Y cells were transfected with Lipofectamine 3000 (Invitrogen), with all transfections performed following the respective manufacturer’s protocols.

### Viral infection

A total of 12 K18-hACE2 Tg mice (strain B6.Cg-Tg(K18-ACE2)2Prlmn/J, Strain #: 034860, The Jackson Laboratory) were intranasally infected with SARS-CoV-2 New York strain at the dose of 2.5 × 10^4^ TCID_50_ (21, 23). Two surviving mice were euthanized on 8 *dpi*. Brain tissues were fixed with formalin and processed for paraffin embedding and sectioning to evaluate the ORF3a protein level and brain tissue damage using IHC compared to controls. The K18-hACE2 mice were housed under pathogen-free conditions, and viral infection was conducted in an animal biosafety level 3 (ABSL-3) facility at the University of Maryland College Park.

### Detection of subcellular location of ORF3a proteins

The procedure to examine the subcellular location of ORF3a proteins has been described previously (21, 72). Briefly, the HA-tagged WT or T223I mutant *ORF3a*-carrying plasmids were transfected into SNB19 cells for 24 h. Immunofluorescent assay was performed by sequential incubation with primary antibodies and Texas red (TR)-labeled secondary antibodies (Vector Laboratories, Burlingame, CA). Mouse anti-HA tag antibody (Santa Cruz, sc7392) detecting HA-tagged ORF3a and rabbit anti-Calnexin antibody (Abcam: ab22595) detecting an ER membrane protein Calnexin were used to visualize co-localization of ORF3a with the ER membrane (22, 72). Cells were examined with a Leica TCS SPII confocal laser scanning system. Two or three channels were recorded simultaneously and/or sequentially and controlled for possible breakthrough between the fluorescein isothiocyanate and Texas red signals and between the blue and red channels. A Fiji image analysis software (https://imagej.net/) that combines ImageJ and JCoP plugin was used to quantify the subcellular location of ORF3a (22, 72). Random images were used for the analyses and the calculation of the mean and standard deviation of Pearson’s correlation coefficients (*P* values).

### Immunostaining brain tissues

Human brain sections were first deparaffinized and then subjected to antigen retrieval in citrate buffer (10 mM citric acid, 0.05% Tween 20, pH 6.0). After blocking sections in blocking buffer (10% donkey serum in 0.2% Triton-X in phosphate-buffered saline (PBS) for 1 h), sections were incubated at 4°C overnight with primary antibodies directed against anti-ORF3a (LS Bio: LS-C829863), anti-SARS-CoV-1/2NP (Sigma-Aldrich: ZMS1075), anti-s100B (Abcam: ab868), anti-GFAP (Sigma-Aldrich: AB5541), anti-TNF (Santa Cruz Biotechnology: sc-1350), and a custom-made anti-Sur1 antibody (118). After several rinses in PBS, sections were incubated with species-appropriate fluorescent secondary antibodies (Alexa Fluor 488 and 555, Molecular Probes, Thermo Fisher Scientific, Waltham, MA, USA) for 1 h at room temperature. Controls for IHC included the omission of primary antibodies.

Immunostaining of mouse brain tissues with the indicated antibodies used in this study has been previously described (41). For immunolabeling of mouse brain tissues, briefly, brains from C57BL/6 or K18-hACE2 transgenic mice and WT controls were fixed in formalin and embedded in paraffin. Sections, 10 µM thick, were cut from the paraffin-embedded blocks containing the entire brain using a microtome. These sections were deparaffinized and rehydrated through graded ethanol (100, 95, and 70%) and xylene washes. Following antigen retrieval, sections were blocked in 5% donkey serum in PBS with 0.2% Triton-X. Unbiased assessments of specific labeling of each image were obtained using NIS-Elements AR software (Nikon Instruments, Melville, NY, USA) from sections immunolabeled as a single batch. All images for a given signal were captured using uniform parameters of magnification, area, exposure, and gain.

### Measurement of *Abcc8*/Sur1 promoter activity

To assess transcriptional activity of *Abcc8*/Sur1, an *Abcc8* luciferase promoter assay was conducted based on our previous report (57). Briefly, 1.0 × 10^4^ SNB19 cells were seeded into individual wells of a white 96-well plate and incubated overnight. For the assay, pCAG-ORF3a carrying plasmid was co-transfected with pGL3-*Abcc8* plasmid or PGL3-basic as the Vec control. Alternatively, SNB19 cells were transduced with Ad5-ORF3a and Ad5 control. To quantify luciferase activity, a dual-luciferase reporter assay system (Promega: Cat# E1910) was used with measurements taken using the Synergy H1 microplate reader (Agilent) at specified time intervals. Signal normalization was achieved using Renilla luciferase as an internal control. Fold changes were calculated relative to the control containing an empty vector.

### Measurement of Sur1-mediated Ca^2+^ influx

To measure the effect of WT or T223I ORF3a on Sur1-regulated ion channel activities, Ca^2+^ influx was measured in two different ways using the Fluo-8 Calcium Assay Kit (AAT Bioquest) following the manufacturer’s instruction. First, Ca^2+^ influx was measured in Dox-inducible SNB19 cells 48 h post-Dox gene induction. Alternatively, SNB19 cells were transduced with Ad5-ORF3a, and the Ca^2+^ influx was measured on day 5 post-transduction. Ca^2+^ influx was measured by following the manufacturer’s instruction. Specifically, ORF3a-producing SNB19 cells were cultured in a 96-well plate and harvested at the indicated time. After removing the growth medium, add the dye in Fluo-8 AM working solution to the cells and incubate them at room temperature for 30–60 min. The fluorescence intensity changes at indicated time were recorded using the Synergy H1 microplate reader with excitation at 490 nm and emission at 525 nm. The change in fluorescence directly correlates to the influx of calcium ions into the cells. To ensure the observed changes are specific, the fluorescence changes were presented as the fold changes over the vector control.

### Quantitative reverse transcription PCR

The procedure of qRT-PCR has been previously described (21, 22). Briefly, cells were cultured in a six-well plate until they reached approximately 70% confluency. Cells were transfected as described above and collected at the indicated time. Total RNA was extracted using Trizol (Invitrogen, 448706). The extracted RNA was treated with RQ1 RNase-Free DNase (Promega: M6101) to eliminate genomic DNA contamination. Subsequently, the RNA was reverse-transcribed into cDNA using reverse transcriptase (Thermo Fisher: 4311235). Real-time PCR was conducted on a QuantStudio 3 Real-Time PCR System using gene-specific primers and SYBR Green Master Mix (Thermo Fisher: A46109) to detect mRNA expression levels. The amplification conditions included 40 cycles of 95°C for 10 s and 60°C for 30 s, followed by a melting curve analysis. Fold change in mRNA expression was calculated using the 2^−ΔΔCT^ method, with GAPDH mRNA serving as the internal control.

### Measurement of cell growth, viability, apoptosis, and necrosis

These procedures have been used in our laboratory previously (19, 21, 22). Briefly, to measure cell growth, viability, and apoptotic cell death, SNB19, SH-SY5Y, or N2a cells were introduced with pCAG-ORF3a plasmid or Ad5-ORF3a as described. At the indicated time, cell growth was counted using a TC20 automated cell counter (Bio-Rad) to determine the total cell count and the number of non-viable (stained) cells, and cell death was quantified by cell counting and trypan blue staining. Cell proliferation and viability were assessed using the MTT assay at the absorbance of 570 nm using an H1M microplate reader (Agilent). Cell apoptosis and necrosis were measured using a RealTime-Glo annexin V apoptosis and necrosis assay kit (Promega).

### Western blot analysis

It was carried out as we described previously (21, 116). In brief, total proteins were extracted from transfected SNB19 cells at indicated time. Equal amounts of total protein were separated on SDS-PAGE gel by electrophoresis and transferred to a polyvinylidene difluoride (PVDF) membrane (Bio-Rad). Target proteins were detected using anti-ORF3a (LS Bio: LS-C829863), a custom-made anti-Sur1 (118), and mouse anti-GAPDH antibody (cell signaling: 2118).

### Drug treatment

The FDA-approved antidiabetic drug and a Sur1 inhibitor, GBC (#G2539; meets USP testing; Sigma-Aldrich) and the ORF3a inhibitor GL (NSC234419) provided by the Developmental Therapeutics Program of the National Cancer Institute, the National Institutes of Health were used in the drug treatment study. Both drugs were stored in dimethyl sulfoxide (DMSO) as the stock concentrations of 50 mM at 4°C and diluted with DMSO for the indicated drug tests.

### Statistical analysis

Statistical analyses were conducted using Prism 9 software (GraphPad, San Diego, CA, USA). Pairwise *t*-tests or one-way analysis of variance (ANOVA) were employed as appropriate. Statistical significance was defined as *P* < 0.05 at the 95% confidence level. Symbols *, **, ***, and **** were used to denote significance levels: *P* < 0.05, *P* < 0.01, *P* < 0.001, and *P* < 0.0001, respectively.

## SUPPLEMENTAL MATERIAL

Figure S1 mbio.02012-25-s0001.tif Detection of SARS-CoV-2 ORF3a and NCp proteins in postmortem brain tissues from COVID-19 patients.

Figure S2 mbio.02012-25-s0002.tif ORF3a induces transcriptional activation of Sur1 in human neuroblastoma cell line SH-SY5Y, leading to cell death.

Figure S3 mbio.02012-25-s0003.tif ORF3a induces transcriptional activation of Sur1 in mouse neuroblast N2a cell line, leading to cell death.

Figure S4 mbio.02012-25-s0004.tif Effect of GBC and GL on ORF3a-induced Sur1 expression and cell death in SNB19 cells.

Legends mbio.02012-25-s0005.pdf Legends for supplemental figures.

ASM does not own the copyrights to Supplemental Material that may be linked to, or accessed through, an article. The authors have granted ASM a non-exclusive, world-wide license to publish the Supplemental Material files. Please contact the corresponding author directly for reuse.
