# Supplementary figures and images for: COVID-19-associated neuroinflammation and astrocyte death in the brain linked to ORF3a-induced activation of Sur1-mediated ion channels
Source: mBio. 2025 Aug 13;16(9):e02012-25. doi: 10.1128/mbio.02012-25 (PMC12421821; doi:10.1128/mbio.02012-25)

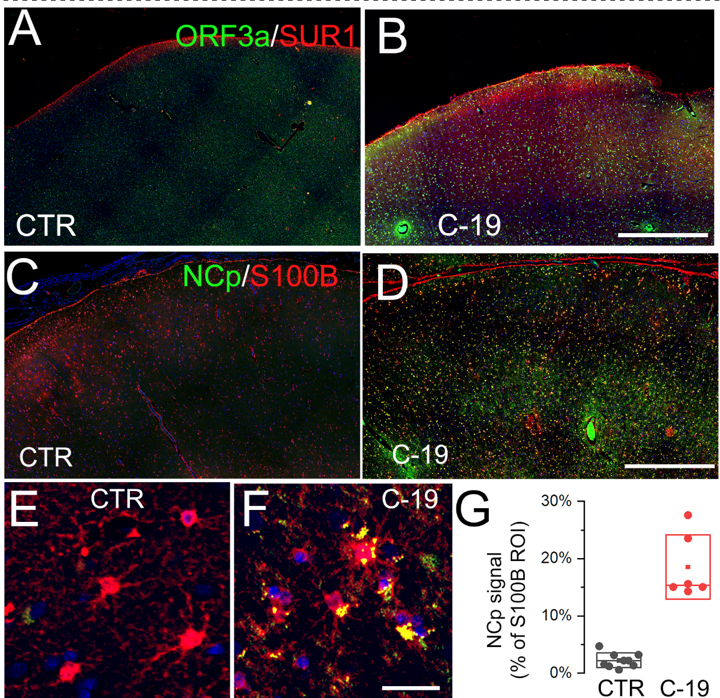

Supplement: Figure S1 — Detection of SARS-CoV-2 ORF3a and NCp proteins in postmortem brain tissues from COVID-19 patients. [file mbio.02012-25-s0001.tif]

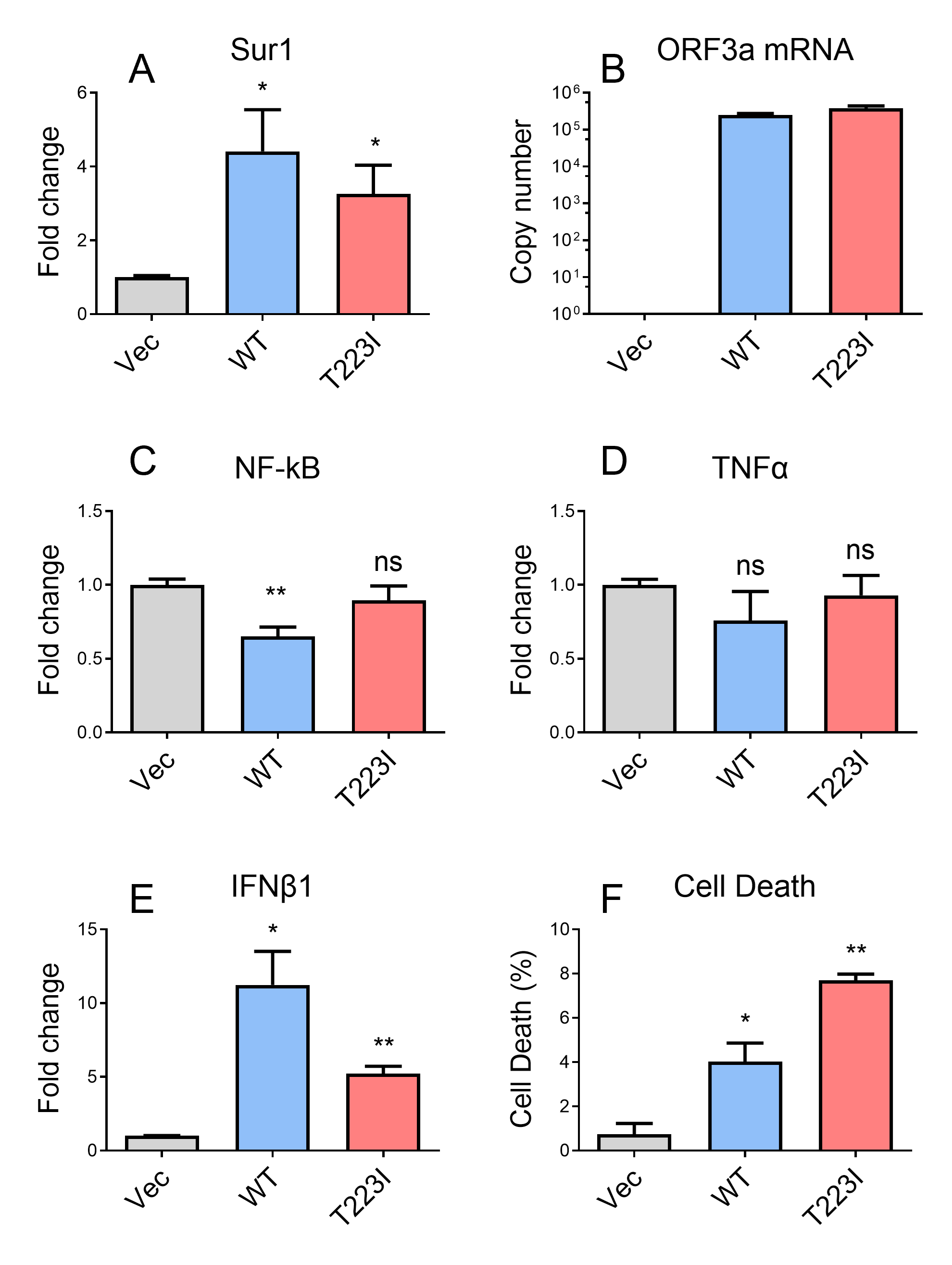

Supplement: Figure S2 — ORF3a induces transcriptional activation of Sur1 in human neuroblastoma cell line SH-SY5Y, leading to cell death. [file mbio.02012-25-s0002.tif]

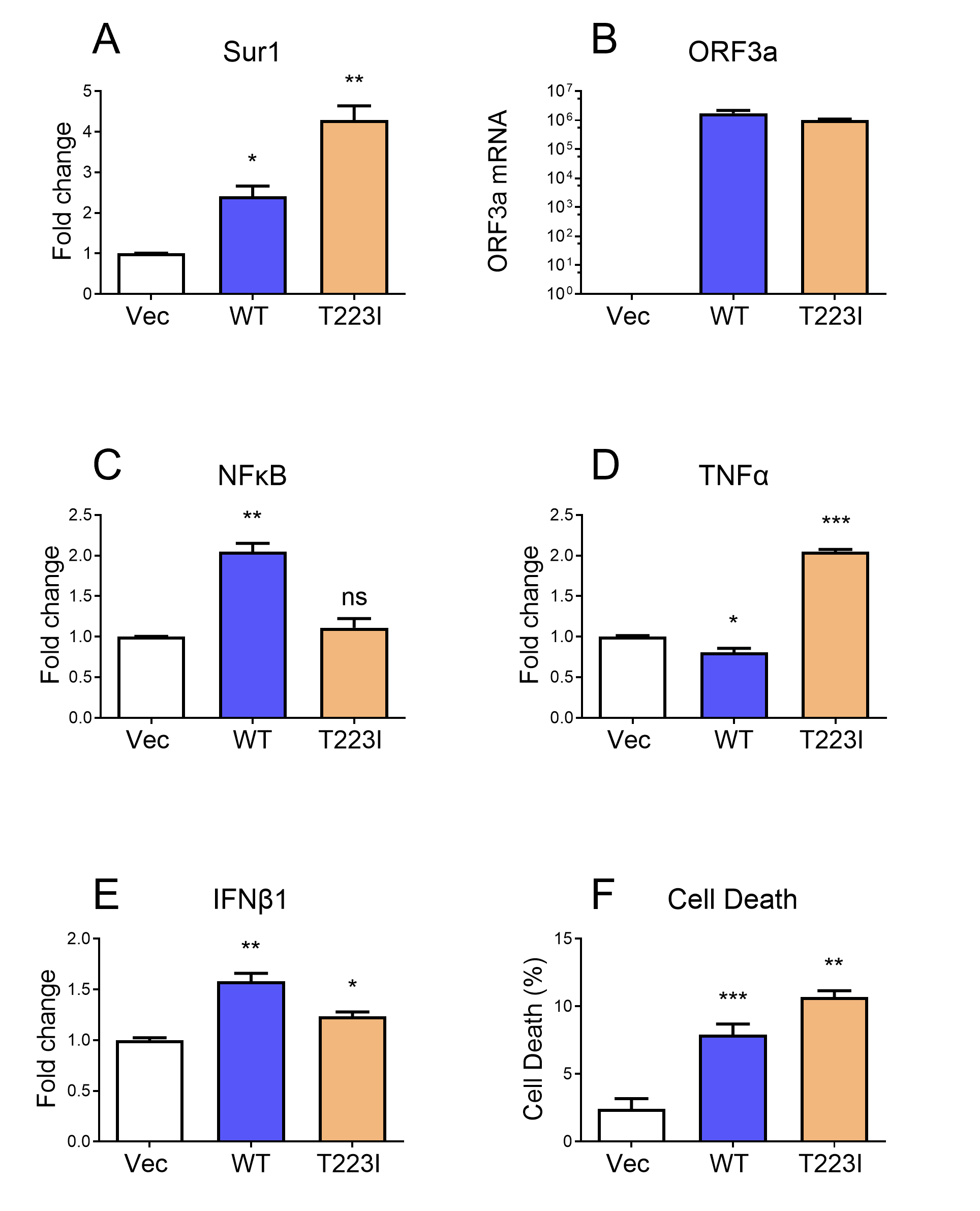

Supplement: Figure S3 — ORF3a induces transcriptional activation of Sur1 in mouse neuroblast N2a cell line, leading to cell death. [file mbio.02012-25-s0003.tif]

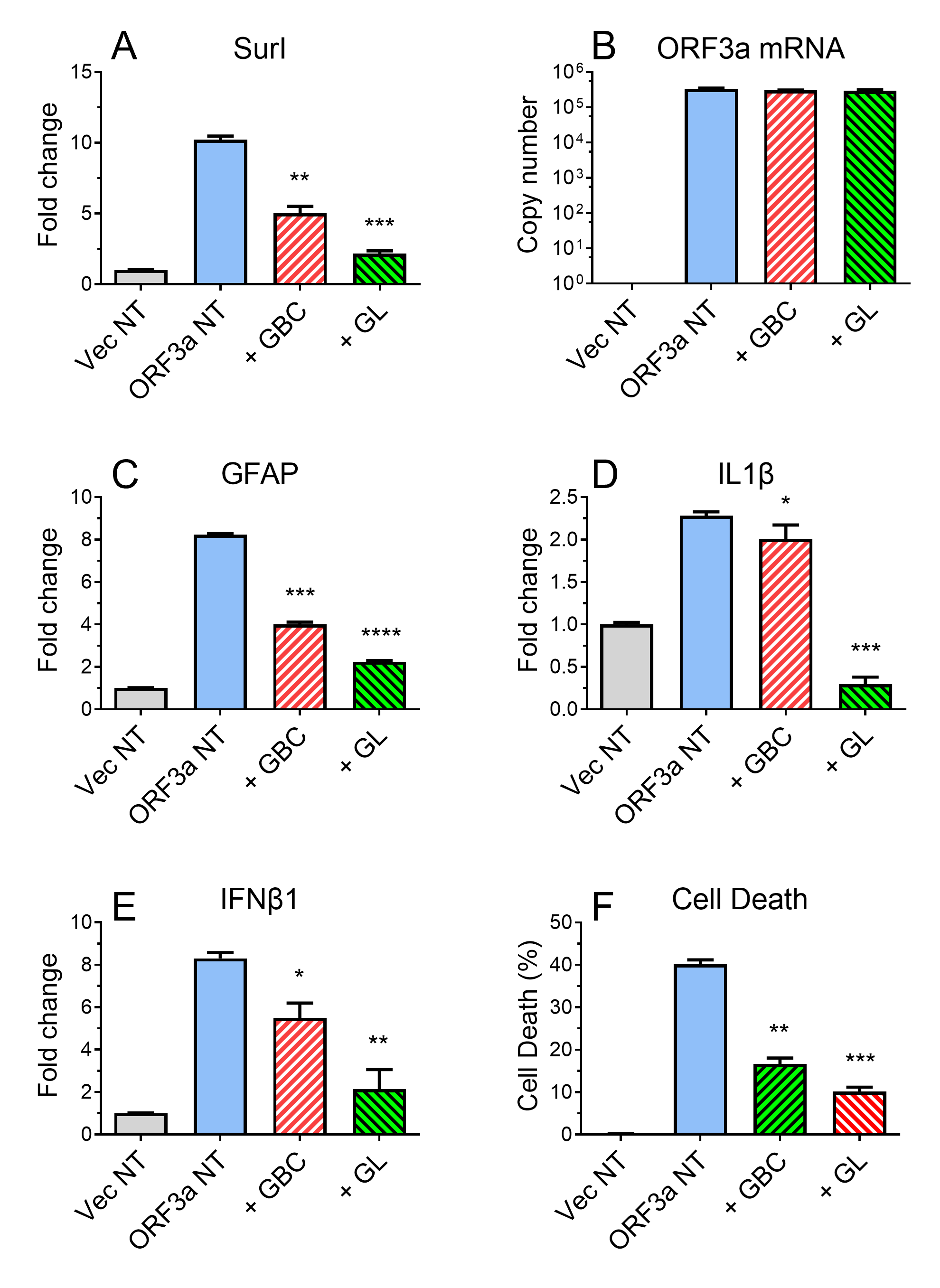

Supplement: Figure S4 — Effect of GBC and GL on ORF3a-induced Sur1 expression and cell death in SNB19 cells. [file mbio.02012-25-s0004.tif]
