# Supplementary material for: COVID-19-associated neuroinflammation and astrocyte death in the brain linked to ORF3a-induced activation of Sur1-mediated ion channels
Source: mBio. 2025 Aug 13;16(9):e02012-25. doi: 10.1128/mbio.02012-25 (PMC12421821; doi:10.1128/mbio.02012-25)
Supplement: Legends — for supplemental figures. [file mbio.02012-25-s0005.pdf]

Figure Legend of Supplemental Figures:

Fig. S1. Detection of SARS-CoV-2 ORF3a and nucleocapsid (NCp) proteins in postmortem brain tissues from COVID-19 patients. (A–G) Co-immunolabeling of ORF3a with SUR1 in cortical gray matter from a control (CTR) subject (**A**, **B**). Images shown are low power views of the same tissues as in **Fig 1A-a**. Data are representative from two control and two COVID-19 (C-19) cases. Co-immunolabeling of NCp with the astrocytic marker S100B in cortical gray matter from a control (CTR) subject (**C**, **E**) and a COVID-19-positive (C-19) patient (**D**, **F**). S100B marks gray matter astrocytes (53). High-magnification images reveal colocalization of the NCp antigen with S100B-positive astrocytes in the C-19 brain tissue (**D**, **F**), which is absent in control tissue (**C**, **E**). (**G**) Quantification of NCp antigen levels in S100B-positive astrocytes. Data were obtained from three 1 × 1 mm regions of interest (ROIs) per brain section from 3 control subjects and 2 COVID-19 patients. Images were acquired using NIS-Elements AR software (Nikon Instruments, Melville, NY, USA) from sections immunolabeled as a single batch. Specific labeling was defined as signal intensity >1.5 × background for S100B and >2 × background for SARS-CoV-2 NCp; S100B-positive ROIs were used for NCp quantification. Scale bars: 1 mm (**A-D**); 50 μm (**E**, **F**).

Fig. S2. ORF3a induces transcriptional activation of Sur1 in human neuroblastoma cell line SH-SY5Y leading to cell death. SH-SY5Y cells were transfected with pCAG plasmids encoding WT or T223I ORF3a. Cells were collected at 24 *hpt*. (**A**) ORF3a induces Sur1 expression. (**B**) Showing both WT and T223I produces high and equivalent levels of mRNA under the same experimental conditions. (**C-D**). ORF3a does not have clear effect on NF-kB and TNFα expression, but it does elevate IFNβ1 (**E**), leading to cell death as measured by Trypan blue exclusion assay (**F**). All markers were measured by qRT-PCR. Statistical significance is indicated as: ns, not significant, * for $p < 0.05$, and ** for $p < 0.01$.

Fig. S3. ORF3a induces transcriptional activation of Sur1 in mouse neuroblast N2a cell line leading to cell death. N2a cells were transfected with pCAG plasmids encoding WT or T223I ORF3a. Cells were collected at 4 - 6 days post-transfection. (**A**) ORF3a induces Sur1 expression. (**B**) Showing both WT and T223I produces high and equivalent levels of mRNA under the same experimental conditions. Both WT and T223I mutant ORF3a show limited effect on NF-kB (**C**) and TNFα (**D**), and IFNβ1 (**E**), leading to cell death as measured by Trypan blue exclusion assay (**F**). All markers were measured by qRT-PCR. Statistical significance is indicated as: ns, not significant, * for $p < 0.05$, ** for $p < 0.01$, and *** for $p < 0.001$.

Fig. S4. Effect of GBC and GL on ORF3a-induced Sur1 expression and cell death in SNB19 cells. The same methods described in **Fig. 6** were used here except the concentration of GBC and GL used were 80 μM and 160 μM, respectively. Statistical significance is indicated as: * for $p < 0.05$, ** for $p < 0.01$, and *** for $p < 0.001$.
